# Evaluation of the Efficacy of LevetiracetamPlus Iron in Comparison With Iron Alone in Controlling and Reducing the Frequency of Breath-Holding Spells in Children Aged 6 Months to 5 Years

**Published:** 2020

**Authors:** Ezatolah ABBASI, Ahad GHAZAVI, Masoud HASSANVAND AMOUZADEH, Mohammad VALIZADEH, Masoud MATINKHAH

**Affiliations:** 1 Pediatric Neurology, Urmia University of Medical Sciences, Urmia, Iran.; 2 Neuroscience Research Center, Qom University of Medical Sciences,Qom,Iran.; 3 Pediatrician, Urmia University of Medical Sciences, Urmia, Iran

**Keywords:** Breath-holding spells, levetiracetam, iron supplements

## Abstract

**Objective:**

A breath-holdingspell (BHS) is defined as an apnea attack following an initial stressful event like anger, sadness, and fear, a painful event like falling or head trauma or any stressful psychology event. This study was designed to assessthe comparative efficacy of levetiracetam plus iron and iron alone in reducingthe BHS frequency in children aged 6 months to 5 years.

**Materials &Method:**

This study was designed asa double-blinded randomized clinical trial. Sixty patients aged 6 months to 5 years were assigned into two groups, withthe first group (A) receiving onlyiron and the second group (B)receiving levetiracetam plus iron. At the end of the study, the efficacy of therapywas analyzed comparatively in these groups.

**Results:**

In this study, the mean number of attacks was 3.94 ± 2.69 before treatment and 1.71 ± 1.99after treatmentin the group A,while it was 6.39 ± 5.7 before treatment and 0.37 ± 1.03after treatment in the group B.The mean number of attacksafter treatment was lower in group B than in group A. In fact, there was a significant difference between the two groups in terms of the number of attacks after treatment (P = 0.003).

**Conclusion:**

Levetiracetam plus iron is more effective than iron alone in reducing BHSs in children aged 6 months to 5 years.

## Introduction

A breath-holdingspell (BHS) is defined asan attack following an initial stressful event such as anger, grief, and fear, a painful stimulus such as falling and head trauma, or any other psychological distress (1, 2). During these attacks, the child starts crying in less than 11 seconds of initial stress, and after a deep inhalation, stops breathing in the exhalation stage, causing facial changes of either cyanosis or pallor. Attacks are sudden and involuntary with benign and self-limited nature, but careful consideration should be given to rule out anyseriousproblem(3, 4).

The age of the onset of BHS is 11.6 months. However, it may occur before 6 months, and in about 11% of cases, the first attack occurs after 2 years of age. Attacks decrease after 2 years of age, and until the age of 4, 11% of children will be attack free;almost all patients will recover by the age of 7. In the majority of articles, the gender of children studied showed a male to female ratio of more than one (5).

The pathophysiology of attacks is a complicated process and is not yet fully discovered. Various studies have been conducted toexaminethe exact cause and the nature of attacks, and each of them presented a separate mechanism. Some studies on the pathophysiologic mechanism suggested that the underlying cause of breath-holdingattacks was the irregularity of the autonomic nervous system (6, 7). In one study on the autonomic nervous system activity,ECG changes such as heart rate, frequency, and length of QT interval during an attackwererecorded.The presence of disorders of the respiratory sinus rhythm and long systolewas noticed during the attack, indicating the presence of a disorder in the regulation of the autonomic nervous system (8).

Iron deficiencyisthe most common cause of anemia in the first 2 years of life, a period which can be observedas the BHS peak in children.Some studies have shown that anemia or low levels of red blood cells may be an etiologic factor,and iron supplements may improve BHS inchildren (9-11).Levetiracetam is a new antiepileptic drug that is structurally similar to piracetam, which has been used in the treatment of BHSs.Previous studies have suggested that piracetam is an anxiety stabilizing agent,and thus,improvesBHSs (12, 13).Levetiracetam has fast and complete digestive absorption,but its protein binding is small. The drug and its metabolites are excreted through the urine and have a half-life of about 6-8 hours. The drugdoes not interact with other antiepileptic drugs, and also, does not affect the pharmacokinetics of other drugs such as contraceptive drugs, digoxin, and warfarin. It is used as an adjunct to control general and partial epilepsy. The exact mechanism of levetiracetamis still unknown, butitsside effects are extremelysmall and do not require any controlled testing(14, 15). According to limited reports regarding the use of new pharmacological methods and the traditional method of using an iron to control BHSs,we designed and implemented this study to evaluate the efficacy of levetiracetamin combination with iron supplementation compared with iron therapy alone in controllingBHSs.

## Materials & Method

In this randomized clinical trial, 60 6-month to 5-year-oldpatients with BHS admitted to the Motahari Hospital of Urmia were enrolledfrom16 Oct 2018 to 18 Feb 2019. The exclusion criteria wereneurodevelopmental delay,any disorder of the nervous system, and discontinuation of medication during the study. 

The patients were randomly assigned into two groups: the first group (A) received only iron supplementat a dose of 5 mg/kg/day (from ferrous sulfate in two divided doses) for threemonthsand the second group(B) started levetiracetam at a dose of 10 mg/kg/day titrating to 40 mg/kg/day (at two doses of CobelDaroucompany) and iron at a dose of 5 mg/kg/day (from ferrous sulfate in two divided doses) for threemonths.All information needed for the research aboutage, sex, and number of attacks before and after treatmentwas gathered monthly through follow-upvisits andovertelephone and entered in checklists for final analysis.The data were analyzed using SPSS version 22 and reported using descriptive statistics (frequency andpercentage) and mean ± standard deviation (mean ± SD). A Student's t-test was used to analyzequantitative data and theChi-square test to analyze thequalitative variables (and Fisher’s exact test if required). The data were also evaluated usingthe Kymograph-Smirnov test. The ANOVA test was used in case the data distribution was normal, and the Mann–Whitney U test wasused to analyze non-parametric data.P-value<0.05 was considered statistically significant.

**Ethical considerations**

Agreement of the University's Ethics Committee was obtained.Ethical considerationsAgreement of Urmia university of medical sciences Ethics Committee was obtained

 (code: IR.UMSU.REC.1397.321).The patients’ dignity was prioritized.All data of the patients were confidential. Conscious informed consent was obtained from the infants’ parents.

## Results

This study was conducted on 60 patients with BHS admittedto the ShahidMotahari Hospital in Urmia from16Oct 2018 to 18 Feb 2019.The patients were divided into two treatment groups:group A with 28 people receiving ferrous sulfate and the group B with 32 patients receiving ferrous sulfate and Levetiracetame(levebel). The mean ages were 19.6 ± 10.32 months and 17.06 ± 8.46 months in the groups A and B, respectively,with the overall mean age being18.25 ± 9.38 months. The mean hemoglobin was 10.86 ± 0.99 in the group A and 10.85 ± 0.87 in the group B, and 10.86 ± 0.92 in total. The mean ferritin was 24.65 ± 7.63, 25.02 ± 5.56,and 24.85 ± 6.55for the group A, the group B, and as the total value,respectively (Tables 1 and 2). There was no significant difference between the two groups regarding age, HB, and ferritin based onthe Mann-Whitney U non-parametric test results (Table 2).

**Table 1:** The mean age, weight, head circumference, Hb, ferritin

		Number		Age	Weight	Head circumference	Hb	Ferritin
Therapeutic group	A	28	Mean	19.60	10.27	45.76	10.86	24.65
			SD	10.32	1.53	1.86	0.99	7.63
			minimum	6	8	43	8/90	12
			maximum	42	14	50	12/90	47
	B	32	Mean	17.06	10.25	45.62	10.85	25.02
			SD	8.46	2.17	1.85	0 .87	5.56
			Minimum	6	6/70	43	8/20	15
			Maximum	47	16	49	12/10	38
P-value				0.381	0.613	0.812	0.682	0.634

**Figure 1:**
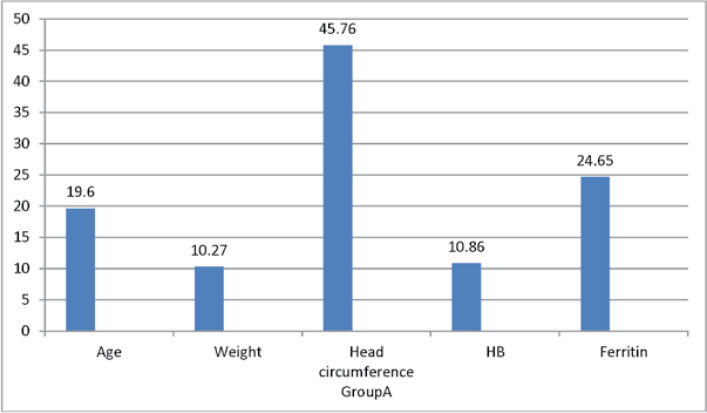
The mean of age, weight, head circumference,Hb, ferritin for Group A

**Figure 2:**
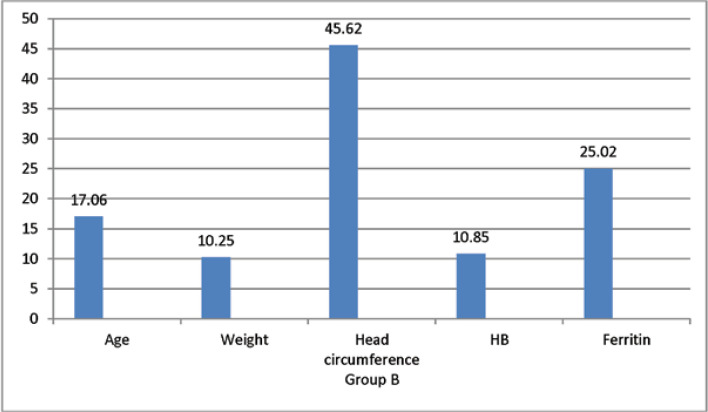
The mean of age, weight, head circumference,Hb, ferritin for Group B

**Table 2:** The mean age, weight, head circumference, Hb, ferritin for all patients

	Age	Weight	Head circumference	Hb	Ferritin	
Mean	18.25	10.26	45.69	10.86	24.85
SD	9.38	1.88	1.84	0.92	6.55
Minimum	6	6.70	43	8.20	12
Maximum	47	16	50	12.90	47
P- Value	0.381	0.613	0.822	0.682	0.634

Group A consisted of 17 boys and 11 girls compared to groupB with 11 boys and 21 girls, making an overall of 28 boys and 32 girls (Table 3-3). There was a significant correlation between the two groups in terms of gender according to the Chi-square test results(P = 0.46); although,itsseverity was low based onthe Cramer's V test results, with the therapeutic effect being higher in the girls.

**Table 3:** Distribution of gender in treatment groups

			Sex		Total	P-Value
			Male	Female		
Group	A	number	17	11	28	0.047
		percentage	60.7%	39.3%	100%	
	B	number	11	21	32	
		percentage	34.4%	65.6%	100%	
Total		number	28	32	60	
		percentage	46.7%	53.3%	100%	

**Figure 3:**
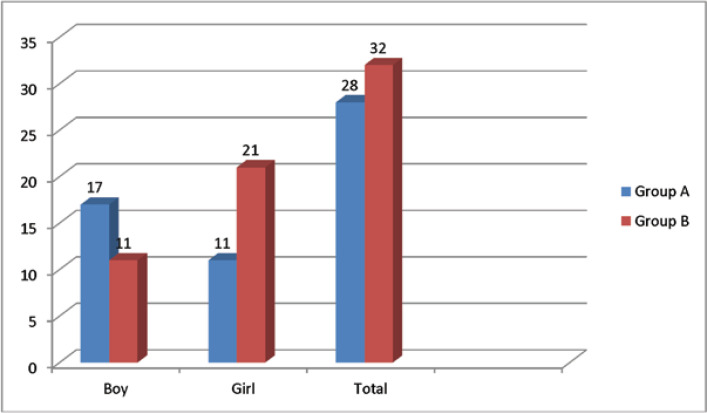
Distribution of gender in the treatment group

The mean number of attacks before and after treatment was 4.39 ± 2.69 and 1.71 ± 1.99in the group A and 6.39 ± 5.07 and 0.37 ± 1.03 in the group B,respectively. The overall mean number of attacks was 4.75 ± 5.75 and 1.68 ± 1.68before and after treatment,respectively (Tables 4 and 5).

**Table 4:** The mean number of attacks

Group		Pre-treatment	Post-treatment
A	number	28	28
	mean	4.39	1.71
	SD	2.69	1.99
B	number	32	32
	mean	6.39	0.37
	SD	5.07	1.03
P-Value		0.072	0.003

**Figure 4:**
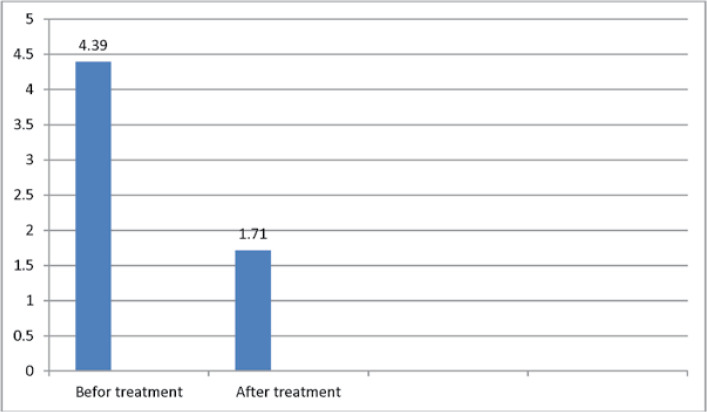
The mean age number of attacks Group A

**Figure 5:**
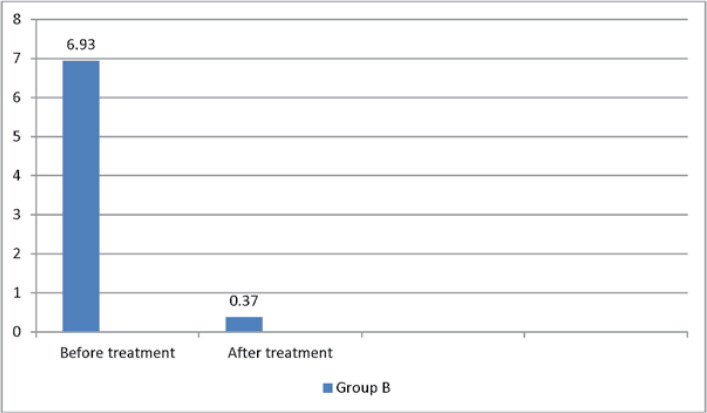
The mean number of attacks in group B

**Figure 6:**
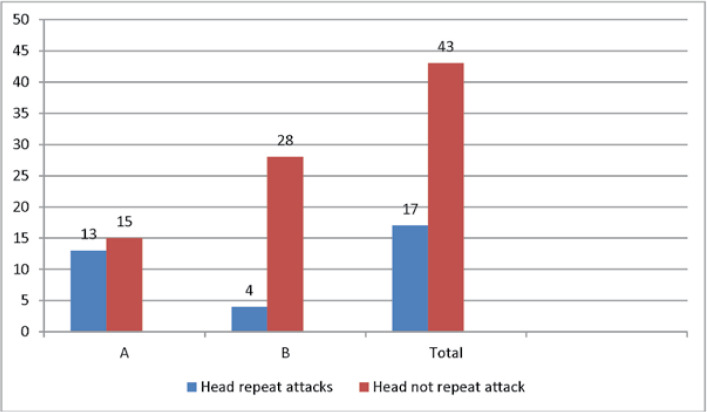
Repeat attacks in different groups

The mean number of attacks was lower in group Bthan in group A. In fact, there was no significant difference between the two groups in terms ofattacks before a treatment based onthe Mann-Whitney U non-parametric test results (P = 0.072). However, there wasa significant difference between the two groups regarding the number of attacks after treatment (P = 0.003). Thirteen(46.4%) and 15(53.6%) patients in group Aexperienced repeated attacks and no attacks compared to four (12.5%) and 28(87.5%)patients ingroup B, respectively. In general, 17 patients(28.3%) experienced repeated attacks, while43 patients (71.7%) did not have further attacks (Table 5, Figure 6).

**Table 5:** Repeat attacks in different groups

			Group		Total
			A	B	
Repeat attacks	Yes	Number	13	4	17
		Percentage	46.4%	12.5%	28.3%
	No	Number	15	28	43
		Percentage	53.6%	87.5%	71.7%
Total		Number	28	32	60
		Percentage	100%	100%	100%

BHS is defined as an attack following an initial stressful event such as anger, grief, and fear, a painful stimulus such as falling and head trauma, or any other psychological distress(1).Iron deficiency isthe most common cause of anemia in the first 2 years of life, a period which can be observedas the BHS peak in children.Some studies have shown that anemia or low levels of red blood cells may be an etiologic factor,and iron supplements may improve BHS in children(9, 10). Levetiracetam is a new antiepileptic drug that is structurally similar to piracetam, which has been used in the treatment of BHSs.Previous studies have suggested that piracetam is an anxiety stabilizing agent, and therefore, improves BHSs (12).Levetiracetam has fast and complete digestive absorption,but its protein binding is small. It is metabolized with thehydrolysis of the enzyme, but is independent of the P450 cytochrome system. The drug and its metabolites are excreted through the urine and have a half-life of about 6-8 hours. The drug does not interact with other antiepileptic drugs, and also, does not affect the pharmacokinetics of other drugs such as contraceptive drugs, digoxin, and warfarin. It is used as an adjunct to control general and partial epilepsy. The exact mechanism of levetiracetamis still unknown, but itsside effects are extremely small and do not require any testing(14).This study was conducted on 60 patients with BHS admittedto the ShahidMotahari Hospital of Urmia to examine the efficacy of the combination of Levebel and iron compared to iron alone in two treatment groups:group A with 28 patientsreceiving only ferrous sulfate and group B with 32 patients receiving ferrous sulfate and Levebel.Group A consisted of 17 boys and 11 girls, and thegroup B consisted of 11 boys and 21 girls, with the overall participantsbeing28 boys and 32 girls.The mean age was 19.60 ± 10.32 monthsin group A and 17.06 ± 8.46 months in group B withthe overall mean age of 18.25 ± 9.38 months. The meannumber of attacks after treatmentwas 37 ± 1.03 in group B and 71 ± 1.99in group A.There was no significant difference between the two groups concerning attacks before a treatment based onthe Mann-Whitney U non-parametric test results (P = 0.072). However, there was a significant difference between the groupsregarding attacks after treatment (P = 0.003).Thirteenpatients(46.4%) in group Aexperiencedrepeated attacks compared to four patients (12.25%) in group B.

Moreover, 15 patients in group A (53.6%) experienced no attacks compared to 28patients(87.5%) ingroup B.In a study conducted by Mocan et al. (2000) in Turkey to assess the effect of iron supplementation on BHSs after three months, there was a significant difference betweeniron-treated children and controls (84.1% vs. 21.4%)regarding the reduction of cyanotic spells. They concluded that treatment for iron deficiency anemia waseffective in reducing the frequency of BHS(16).However, thisis not consistent with the result of our study showinga response rate of only54% in those treated with iron alone.In a study conducted by Azamet al. (2008) in Pakistan, the effect of prophylactic piracetamwas examined on severe cases of BHS. The spells completely disappeared in 81% of children, and the number of attacks decreasedto less than one per month with less severityin 9%of them. Prophylaxis was given for 3 to 6 months (average of 5months). They concluded that piracetam was effective in preventingsevere BHS(17). Considering the similarity of the chemical structure of levotiracetame to that of piracetam, the findings of theirstudy are consistent with those of our study.In another study conducted by Ashraf Zadehet al.(2005) in Iran, the effect of piracetamwas examined on BHS. They reported that 19 (90.5%) of the children receiving piracetam had a good response, while in the group receiving the placebo, onlyeightchildren (40%) experienced noattacks (P = 0.002). The incidence of clinical complications was similar inboth groups. They concluded that piracemwasan effective medication for the treatment of childhood BHS, with no major side effects(18), which isconsistent with the results of our study.Some previously published studiesexamined the effect of piracetam and iron in comparison with iron alone oncontrollingBHS(19, 20). These studies reported a significant difference between the two groups concerning the frequency reduction of attacks (P=0.001), with the mean number of attacks being one spell in a month in the first group and two attacks in the second group. They concluded that treating spells with the combination of piracetam and iron wasmore effective than onlywith iron, which is compatible with the findings of our study.

## In Conclusion

Based on the findings of our study, we suggest that the combination of levetiracetam and iron ismore effective than iron alonein controlling BHSs.Beinga safe pharmaceutical profile in children,levetiracetam can be safely used to control BHS in combination with iron.
